# Is open bone graft always necessary when treating aseptic subtrochanteric nonunion with a reamed intramedullary nail?

**DOI:** 10.1186/s12891-021-04016-y

**Published:** 2021-03-01

**Authors:** Won Chul Shin, Jae Hoon Jang, Nam Hoon Moon, Se Bin Jun

**Affiliations:** 1 Department of Orthopaedic Surgery, Research Institute for Convergence of Biomedical Science and Technology, Pusan National University Yangsan Hospital, Pusan National University School of Medicine, Yangsan, Republic of Korea; 2 Department of Orthopaedic Surgery, Trauma Center, Bio-medical Research Institute, Pusan National University Hospital, 179 Gudeok-Ro, Seo-Gu, Busan, 49241 Republic of Korea; 3 Department of Orthopaedic Surgery, Bio-medical Research Institute, Pusan National University Hospital, Busan, Republic of Korea

**Keywords:** Subtrochanteric fracture, Nonunion, Intramedullary nail, Bone graft

## Abstract

**Background:**

This study aimed to compare the radiological results between closed nailing without bone graft (BG) and open nailing with BG for aseptic subtrochanteric nonunion and to determine when an open procedure with BG should be considered.

**Methods:**

In this retrospective study, we investigated patients who underwent surgical intervention for subtrochanteric nonunion between January 2008 and March 2018 in two institutions. Patients with infection, large bone defect, pathologic fracture, open fracture, previous surgery using plate, and follow-up of less than 1 year were excluded. We compared the demographic details and radiological results between patients who underwent the open procedure with BG (BG group) and the closed procedure without BG (non-BG group) as a historical control, and risk factors for the failure of revision surgery were evaluated.

**Results:**

Thirty-seven patients met the criteria and were divided into the following two groups: the BG group (n=19) who underwent open nailing with BG and the non-BG group (n=18) who underwent closed reamed nailing without BG. The mean degrees of correction of varus and flexion deformity were significantly different (p=0.001, respectively), 6.2° and 2.9° in the BG group and 4.1° and 0.6° in the non-BG group, respectively. Bony union was observed in 17 cases (89.5%) in an average of 7.4 months in the BG group and in 16 cases (88.9%) in 7.6 months in the non-BG group, with no significant differences. The factors that were significantly associated with failure of revision were atypical fracture, two or more previous surgeries, and varus and sagittal anterior angulation.

**Conclusions:**

The radiological results of closed reamed nailing without BG for subtrochanteric nonunion were satisfactory. In the effort of percutaneous realignment, gap reduction, and intramedullary reaming, the radiological results of closed nailing without BG were not different from those of open nailing with BG; therefore, closed procedure without BG may be an acceptable option in appropriately selected patients.

## Background

Surgical treatment for subtrochanteric nonunion commonly requires realignment of residual deformity, mechanical stability, and improvement of the biological environment around the fracture site [1–5]. Autogenous bone graft (BG) is frequently considered when planning surgical treatment for subtrochanteric nonunion because of the disruption of the biological environment by previous open surgery and fracture gap by insufficient reduction [6, 7]. However, determining the extent of compromised biology and when BG should be considered is still unclear. Furthermore, BG is performed in an open manner concurrently with the correction of malalignment; hence, these open procedures might cause additional damage to biology, require extra effort and surgical time, and result in donor site morbidity. Therefore, deciding whether to perform BG is challenging. Hence, this study aimed to determine the results of surgical treatment without open BG for subtrochanteric nonunion assuming that intramedullary reaming might work as internal BG [8–10].

The authors in this study hypothesized that closed procedures, including percutaneous correction of malalignment, fracture gap reduction, and reamed nailing without BG represent similar outcomes to those of open procedure, including open realignment and nailing with BG. This study aimed to compare the radiological results of surgery for aseptic subtrochanteric nonunion between the closed procedure without BG and the open procedure with BG and to determine when the open procedure with BG should be considered.

## Methods

### Study population

This retrospective study conformed to the Declaration of Helsinki and the institution’s Good Clinical Practice guidelines and was approved by the institutional review board and registered with ClinicalTrials.gov (NCT04651647). We investigated the medical records and radiographs of patients who underwent surgical intervention using intramedullary nailing for subtrochanteric nonunion between January 2008 and March 2018 in two institutions. Cases with infection, large bone defect, pathologic fracture, open fracture, previous and revision surgery using plate, and follow-up of less than 1 year were excluded. All patients had intramedullary nails in place and developed aseptic nonunion. Septic nonunion was ruled out through history taking, clinical examination, and tests for blood inflammatory markers preoperatively [5, 11].

Two surgeons performed all surgeries at the two institutions. The closed procedure without BG was performed consecutively between January 2015 and March 2018 with the expectation of similar outcomes to the open procedure with BG. There was no specific indication for the selection of each surgical method. The open procedure with BG was performed for the rest of the period. Thus, we compared the demographic details and radiological results between patients who underwent the open procedure with BG (BG group) and patients who underwent the closed procedure without BG (non-BG group) as a historical control.

### Surgical procedure

**Fig. 1:**
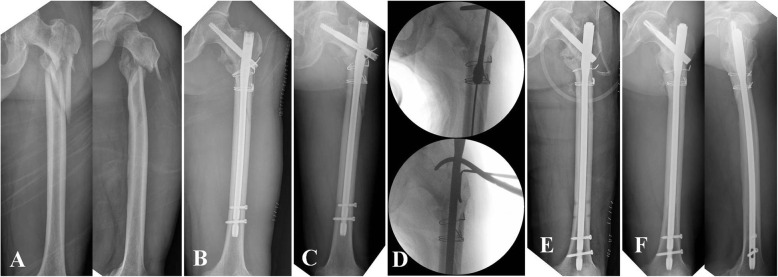
**a** A 52- year-old man who sustained reverse oblique fracture with subtrochanteric extension by high-energy injury. **b** Postoperative radiograph after open surgery. **c** Nonunion and implant failure at 14 months after initial surgery. **d** Revision surgery using closed procedure without bone graft. **e** Postoperative radiograph. **f** Bony union at 7 months after revision surgery

In the non-BG group, all surgeries were performed with patients assuming a supine position with a bump under the involved buttock on a radiolucent, ordinary operating table under general anesthesia. All procedures were performed under image intensifier control. The previous implant was removed through the previous incision. Implants that could not be removed without using an additional open approach, such as wires, were left unchanged. Moreover, residual varus and/or flexion deformity was corrected percutaneously or through existing incision using various instruments, including ball spike pusher, kidney clamp, and Schanz pin. After achieving satisfactory reduction, a new entry point was established, which was positioned more posteromedially as necessary; subsequently, reaming was performed. If a new entry point was not established, we reamed the entry canal more posteromedially using a rigid drill reamer through the protection sleeve. Reaming was performed at the fracture site sufficiently using a rigid drill reamer or flexible reamer, according to the fracture level. A nail as large in diameter as possible was inserted with 1.0–1.5mm of overreaming. After inserting a cephalomedullary screw or blade, we reduced the fracture gap using a forward-striking technique [12], and subsequently, distal interlocking screws were inserted after confirming angular and rotational alignment. Depending on the previous implant, a reconstructive nail (A2FN; DePuy Synthes, Oberdorf, Switzerland) or long proximal femoral nail (PFNA II; DePuy Synthes, Oberdorf, Switzerland) was selected for the new implant according to the surgeon’s judgement. The surgeons attempted to select a new nail to avoid the position of the previous cephalomedullary device and enhance stability by purchasing the device in sound bone stock depending on the type and position of the previous device. During all procedures, the fracture site was not exposed, except for a stab incision for percutaneous manipulation and incision for the removal and insertion of previous and new implants (Fig. 1).

In the BG group, most procedures, sequences, and selections of new implants were similar to those in the non-BG group, except for exposure of the fracture site, additional implant removal (such as wire), debridement of the nonunion site, soft tissue release for the open reduction, and autogenous BG harvested from the iliac crest of the available side.

Tissue samples for microbiology analysis from the reaming debris in the proximal entry in the non-BG group and from debridement of the nonunion site in the BG group were collected to rule out the presence of hidden low-grade infection. After collection of the samples, prophylactic antibiotics were administered according to our institutional protocol.

### Assessment of measures

Demographic details, including age, gender, body mass index, type of initial trauma (low- or high-energy injury), AO/OTA fracture classification [13], atypical femoral fracture, implant at the previous surgery, number and types (open or closed) of previous surgeries, and implant failure were recorded for comparison between the two groups. Atypical femoral fracture was diagnosed using initial plain radiographs according to the American Society of Bone and Mineral Research criteria [14]. Time to revision, implant at revision, and follow-up period were reviewed to compare the postoperative data between the two groups.

**Fig. 2:**
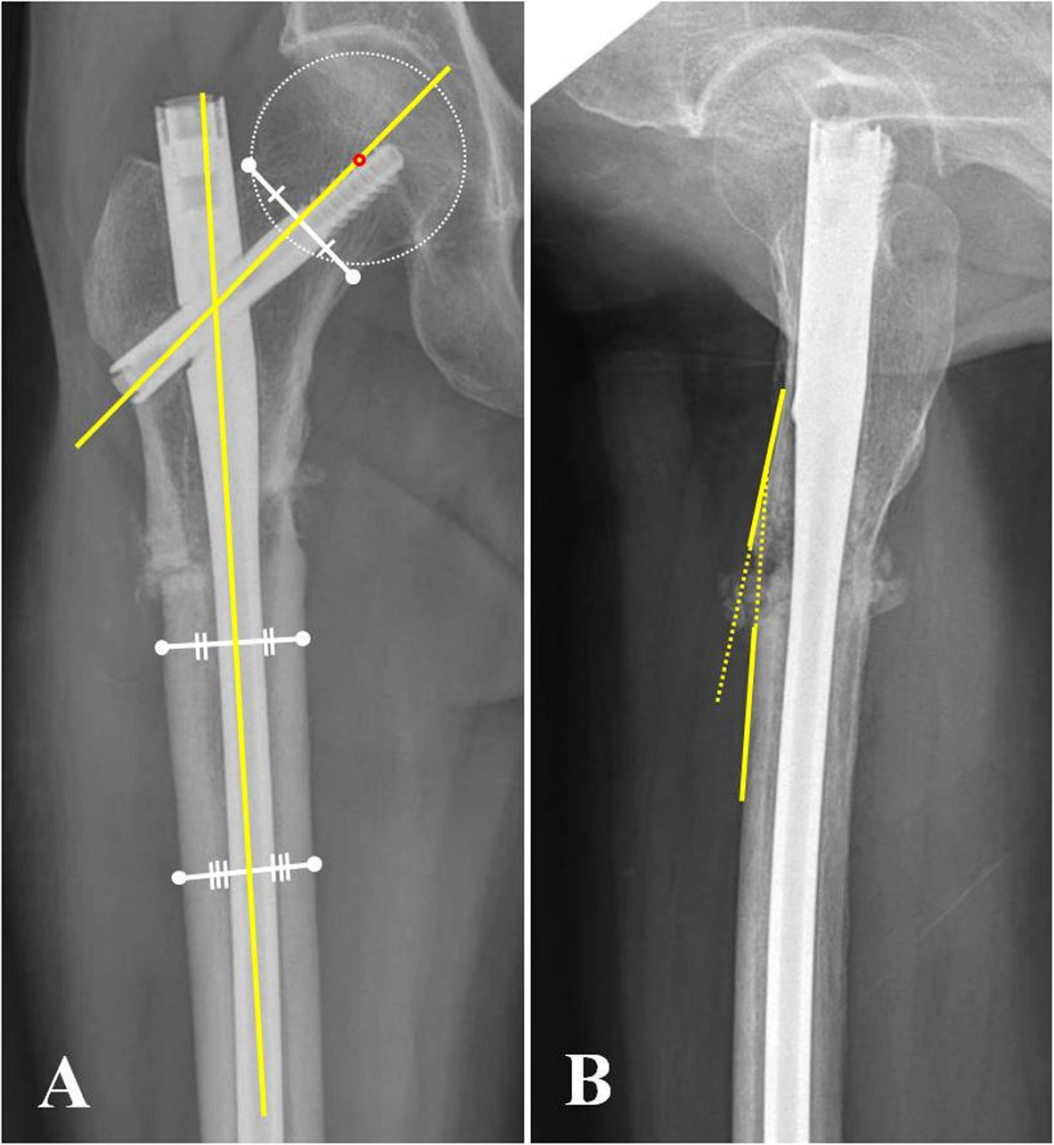
Radiographic measurements of the neck-shaft angle (**a**) at the anteroposterior radiograph and sagittal anterior angulation (**b**) at the lateral radiograph

Alignment before and after revision, contralateral neck-shaft angle, the degree of correction of malalignment after revision, union, and time to union were measured for radiological evaluation and comparison between the two groups. The correction of malalignments and postoperative alignments according to the new implants with different designs (reconstructive nails and long proximal femoral nails) were also investigated and compared. Alignments included the neck shaft angle in the anteroposterior (AP) radiograph, sagittal anterior angulation in the lateral radiograph, and fracture gap that was measured using the greatest distance between the proximal and distal major fragments in either the AP or lateral radiographs (Fig. 2). The degree of correction of malalignment was the difference between the angulations before and after revision in the three alignments. Union was defined as painless full weight bearing with the absence of a fracture line or bridging callus across at least three cortices on the AP and lateral views. All radiographical measurements in this study were conducted by an orthopaedic surgeon who is also one of the authors.

### Statistical analysis

The Mann-Whitney U test was used to compare the variables of age, body mass index, alignments before and after revision, the degree of correction of malalignment after revision, time to revision, follow-up period, and time to union. Fisher’s exact test or chi-squared test was used to compare the variables of gender, type of trauma, fracture classification, atypical femoral fracture, affected side, the number and type of previous surgery, implant at initial and revision surgery, implant failure, and union. Risk factors for the failure of revision surgery were evaluated by univariate analysis using Fisher’s exact test or chi-squared test for categorical parameters and binary logistic regression for continuous parameters. SPSS software (version 22.0; SPSS Inc., Chicago, IL, USA) was used for all statistical analyses. P≤0.05 was considered statistically significant.

## Results

**Fig. 3:**
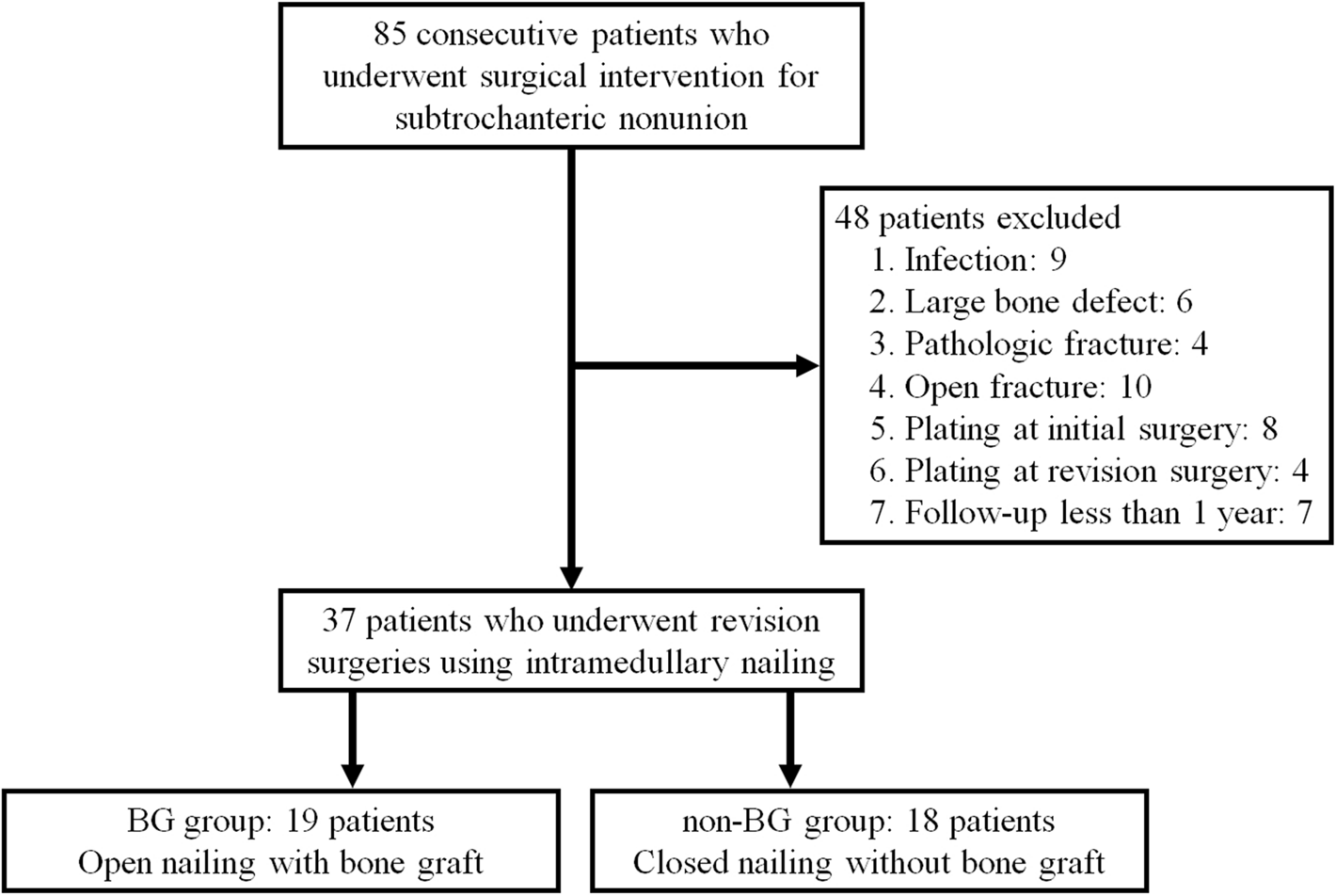
Flowchart of the study design

Thirty-seven patients met the criteria, and they were divided into the following two groups: the BG group who had undergone the open procedure with BG and the non-BG group who had undergone the closed procedure without BG (Fig. 3). The cultures of the tissue samples collected during the revision surgery did not reveal any organism, and there was no significant evidence of infection on serial laboratory examination (white blood cell count, C-reactive protein, and erythrocyte sedimentation rate) after the surgery in any of the patients in the two groups. The demographic characteristics of the study participants in each group and comparison between the groups are presented in Table 1. The BG and non-BG groups included 19 and 18 patients with mean ages of 60.9 years (39–81 years) and 55.8 years (32–82 years), respectively. Gender and affected side were significantly different between the two groups; however, none of the other demographic details were significantly different between the two groups. There were three and two atypical femoral fractures in each group. Five patients in the BG group and two in the non-BG group had two or three surgeries before revision. The mean neck-shaft angle, sagittal anterior angulation, and fracture gap before revision were 118.1° (113–125), 5.7° (2.0–15.0), and 4.7mm (2.0–8.0) in the BG group, 119.7° (115–126), 4.2° (1.0–10.0), and 4.3mm (2.0–8.0) in the non-BG group, respectively.

**Table 1:** Demographics of study participants and comparison between the groups

Variables	BG group	Non-BG group	*p* value
Number	19 (51.4)	18 (48.6)	-
Age (years)	60.9 ± 10.3 (39–81)	55.8 ± 14.9 (32–82)	0.280
Gender (male)	8 (42.1)	14 (77.8)	0.027
Body mass index (kg/m^2^)	24.5 ± 2.3 (18.7–27.3)	24.3 ± 2.6 (20.8–28.9)	0.221
Type of trauma (high energy)	11 (57.9)	11 (61.1)	0.842
Fracture classification			0.665
31A3 (extension to subtrochanteric region)	3	3	
32A1, A2, A3	9	7	
32B1, B2, B3	7	8	
Atypical femoral fracture	3 (15.8)	2 (11.1)	1.000
Affected side (right)	13 (68.4)	5 (27.8)	0.013
Implant at initial surgery			0.694
Conventional IM nail	5 (26.3)	4 (22.2)	
Reconstructive IM nail	2 (10.5)	1 (5.6)	
Short PFN	5 (26.3)	3 (16.7)	
Long PFN	7 (36.8)	10 (55.6)	
The number of previous surgery			0.151
1	14 (73.7)	16 (88.9)	
2	5 (26.3)	1 (5.6)	
3	0 (0.0)	1 (5.6)	
Previous open surgery	16 (84.2)	13 (72.2)	0.447
Implant failure	9 (47.4)	12 (66.7)	0.236
Alignment before revision			
Neck-shaft angle (°)	118.1 ± 3.6 (113–125)	119.7 ± 2.7 (115–126)	0.061
Sagittal anterior angulation (°)	5.7 ± 3.2 (2.0–15.0)	4.2 ± 2.5 (1.0–10.0)	0.150
Fracture gap (mm)	4.7 ± 1.4 (2.0–8.0)	4.3 ± 1.6 (2.0–8.0)	0.306
Contralateral neck-shaft angle (°)	128.1 ± 2.1 (125.0–133.0)	127.2 ± 1.4 (125.0–130.0)	0.199

Values are presented as mean ± standard deviation (range), or number (%)

*BG* bone graft, *IM* intramedullary, *PFN* proximal femur nail

Postoperative radiological outcomes in each group and comparison between the two groups are presented in Table 2. After revision, the mean degrees of correction in varus and sagittal angulation and fracture gap reduction were 6.2° (0–9.0), 2.9° (0–12.0), and 2.9mm (1.0–6.0) in the BG group and 4.1° (0–6.0), 0.6° (0–2.0), and 2.6mm (1.0–6.0) in the non-BG group, respectively. There were significant differences in the correction of varus and sagittal angulation between the two groups (p=0.001, respectively). Bony union was observed in 17 cases (89.5%) in an average of 7.4 months (6.0–10.0) in the BG group and in 16 cases (88.9%) in the 7.6 months (6.0–9.0) in the non-BG group, with no differences between the two groups. No significant differences in the correction of malalignments and postoperative alignments were observed between the two different implants (Table 3).

**Table 2:** Postoperative radiological results of study participants and comparison between the groups

Variables	BG group (*n* = 19)	Non-BG group (*n* = 18)	p value
Time to revision (months)	16.2 ± 5.7 (9.0–30.0)	15.7 ± 7.6 (9.0–43.0)	0.781
Implant at revision			0.197
Reconstructive IM nail	11 (57.9)	14 (77.8)	
Long PFN	8 (42.1)	4 (22.2)	
Correction of malalignment after revision			
Varus correction (°)	6.2 ± 2.5 (0–9.0)	4.1 ± 1.9 (0–6.0)	0.001
Sagittal correction (°)	2.9 ± 3.0 (0–12.0)	0.6 ± 0.7 (0–2.0)	0.001
Gap reduction (mm)	2.9 ± 1.3 (1.0–6.0)	2.6 ± 1.2 (1.0–6.0)	0.324
Alignment after revision			
Neck-shaft angle (°)	124.2 ± 2.1 (120.0–130.0)	123.8 ± 1.8 (121.0–127.0)	0.374
Difference from contralateral side (°)	3.8 ± 1.2 (2.0–6.0)	3.3 ± 1.3 (1.0–6.0)	0.258
Sagittal anterior angulation (°)	2.8 ± 1.0 (1.0–5.0)	3.6 ± 1.9 (1.0–8.0)	0.199
Fracture gap (mm)	1.8 ± 0.6 (1.0–3.0)	1.7 ± 0.7 (1.0–3.0)	0.753
Follow-up period (months)	18.1 ± 5.0 (12.0–30.0)	18.9 ± 7.6 (12.0–43.0)	0.988
Union	17 (89.5)	16 (88.9)	1.000
Time to union (months)	7.4 ± 1.3 (6.0–10.0)	7.6 ± 0.9 (6.0–9.0)	0.654

Values are presented as mean ± standard deviation (range), or number (%)

*BG* Bone graft, *IM* intramedullary, *PFN* proximal femur nail

**Table 3:** Postoperative radiological results of study participants and comparison between the new implants

Variables		Reconstructive IM nail	Long PFN	*p* value
BG group (*n* = 19)	Number	11	8	
	Correction of malalignment after revision			
	Varus correction (°)	6.2 ± 2.4 (0–9.0)	6.1 ± 2.7 (0.0–8.0)	0.840
	Sagittal correction (°)	3.2 ± 3.7 (0–12.0)	2.5 ± 1.6 (0.0–4.5)	0.904
	Gap reduction (mm)	3.0 ± 1.6 (1.0–6.0)	2.9 ± 0.6 (2.0–4.0)	0.968
	Alignment after revision			
	Neck-shaft angle (°)	124.5 ± 2.5 (120.0–130.0)	123.9 ± 1.4 (122.0–125.0)	0.840
	Difference from contralateral side (°)	3.9 ± 1.2 (2.0–6.0)	3.8 ± 1.3 (2.0–6.0)	0.778
	Sagittal anterior angulation (°)	2.5 ± 0.9 (1.0–4.0)	3.1 ± 1.2 (1.0–5.0)	0.310
	Fracture gap (mm)	1.7 ± 0.6 (1.0–3.0)	1.9 ± 0.6 (1.0–3.0)	0.657
Non-BG group (*n* = 18)	Number	14	4	
	Correction of malalignment after revision			
	Varus correction (°)	3.9 ± 2.1 (0.0–6.0)	4.8 ± 1.5 (3.0–6.0)	0.505
	Sagittal correction (°)	0.4 ± 0.6 (0.0–2.0)	1.1 ± 0.9 (0.0–2.0)	0.158
	Gap reduction (mm)	2.5 ± 1.4 (1.0–6.0)	2.8 ± 0.5 (2.0–3.0)	0.574
	Alignment after revision			
	Neck-shaft angle (°)	123.7 ± 1.8 (121.0–127.0)	124.3 ± 2.1 (122.0–127.0)	0.574
	Difference from contralateral side (°)	3.4 ± 1.1 (2.0–5.0)	3.3 ± 2.2 (1.0–6.0)	0.798
	Sagittal anterior angulation (°)	3.4 ± 1.6 (1.0–7.0)	4.3 ± 3.0 (1.0–8.0)	0.645
	Fracture gap (mm)	1.6 ± 0.6 (1.0–3.0)	2.0 ± 0.8 (1.0–3.0)	0.442
All participants (*n* = 37)	Number	25	12	
	Correction of malalignment after revision			
	Varus correction (°)	4.9 ± 2.4 (0.0–9.0)	5.7 ± 2.4 (0.0–8.0)	0.296
	Sagittal correction (°)	1.7 ± 2.8 (0.0–12.0)	2.0 ± 1.5 (0.0–4.5)	0.095
	Gap reduction (mm)	2.7 ± 1.5 (1.0–6.0)	2.8 ± 0.6 (2.0–4.0)	0.491
	Alignment after revision			
	Neck-shaft angle (°)	124.0 ± 2.1 (120.0–130.0)	124.0 ± 1.5 (122.0–127.0)	0.786
	Difference from contralateral side (°)	3.6 ± 1.2 (2.0–6.0)	3.6 ± 1.6 (1.0–6.0)	0.987
	Sagittal anterior angulation (°)	3.0 ± 1.4 (1.0–7.0)	3.5 ± 1.9 (1.0–8.0)	0.511
	Fracture gap (mm)	1.7 ± 0.6 (0.0–12.0)	1.9 ± 0.7 (1.0–3.0)	0.360

Values are presented as mean ± standard deviation (range), or number (%)

*IM* intramedullary, *PFN* proximal femur nail, BG bone graft

Factors associated with failure of revision surgery by univariate analysis are presented in Table 4. Statistical significance was observed in cases of atypical femoral fracture (odds ratio [OR], 10.0; 95% confidence interval [CI], 1.011–98.876), two or more previous surgeries (OR, 21.750; 95% CI, 1.798–263.106), varus (OR, 3.221; 95% CI, 1.042–9.959), and sagittal anterior angulation (OR, 2.653; 95% CI, 1.196–5.886).

**Table 4:** Univariate analysis

Variables	All	Union (*n* = 33)	Non-union (*n* = 4)	*p* value	Odds Ratio (95% CI)
Age (years)	58.4 ± 2.4 (32.0–82.0)	57.8 ± 13.4 (32.0–82.0)	63.5 ± 3.7 (59.0–68.0)	0.400	1.041 (0.948–1.142)
Gender					
Female	15 (40.5)	12 (36.4)	3 (75.0)	0.171	Reference
Male	22 (59.5)	21 (63.6)	1 (25.0)		0.190 (0.018–2.041)
BMI (kg/m^2^)	24.4 ± 2.4 (18.7–28.9)	24.4 ± 2.4 (18.7–28.9)	24.5 ± 2.9 (21.9–27.1)	0.914	1.024 (0.664–1.581)
Affected side					
Right	18 (48.6)	16 (48.5)	2 (50.0)	0.954	Reference
Left	19 (51.4)	17 (51.5)	2 (50.0)		0.941 (0.118–7.499)
Type of Trauma					
Low-energy	15 (40.5)	13 (39.4)	2 (50.0)	0.685	Reference
High-energy	22 (59.5)	20 (60.6)	2 (50.0)		0.650 (0.081–5.206)
Fracture classification					
31A3	6 (16.2)	6 (18.2)	0 (0.0)	0.053	Undefined
32A1, A2, A3	16 (43.2)	12 (36.4)	4 (100.0)		
32B1, B2, B3	15 (40.5)	15 (45.5)	0 (0.0)		
Atypical femoral fracture					
No	32 (86.5)	30 (90.9)	2 (50.0)	0.049	Reference
Yes	5 (13.5)	3 (9.1)	2 (50.0)		10.0 (1.011–98.876)
Type of previous surgery					
Closed	8 (21.6)	8 (7.1)	0 (0.0)	0.557	Undefined
Open	29 (78.4)	25 (75.8)	4 (100.0)		
The number of previous surgery					
1	30 (81.1)	29 (87.9)	1 (25.0)	0.015	Reference
2 or 3	7 (18.9)	4 (6.2)	3 (75.0)		21.750 (1.798–263.106)
Time to revision (months)	15.9 ± 6.6 (9.0–43.0)	15.0 ± 4.8 (9.0–30.0)	23.8 ± 13.7 (14.0–43.0)	0.052	1.149 (0.999–1.322)
Bone graft					
No	18 (48.6)	16 (48.5)	2 (50.0)	0.954	Reference
Yes	19 (51.4)	17 (51.4)	2 (50.0)		0.941 (0.118–7.499)
Implant					
Reconstructive IM nail	25 (67.6)	23 (69.7)	2 (50.0)	0.582	Reference
Long PFN	12 (32.4)	10 (30.3)	2 (50.0)		2.300 (0.283–18.705)
Varus angulation (°)	3.6 ± 1.3 (1.0–6.0)	3.4 ± 1.2 (1.0–6.0)	5.0 ± 1.4 (3.0–6.0)	0.042	3.221 (1.042–9.959)
Sagittal anterior angulation (°)	3.2 ± 1.6 (1.0–8.0)	2.9 ± 1.2 (1.0–6.0)	5.5 ± 2.6 (2.0–8.0)	0.016	2.653 (1.196–5.886)
Fracture gap (mm)	1.8 ± 0.6 (1.0–3.0)	1.6 ± 0.5 (1.0–2.0)	3.0 ± 0.0 (3.0–3.0)	1.000	Undefined

Values are presented as mean ± standard deviation (range), or number (%)

BMI body mass index, IM intramedullary, *PFN* proximal femur nail, *CI*, confidence interval

## Discussion

**Fig. 4:**
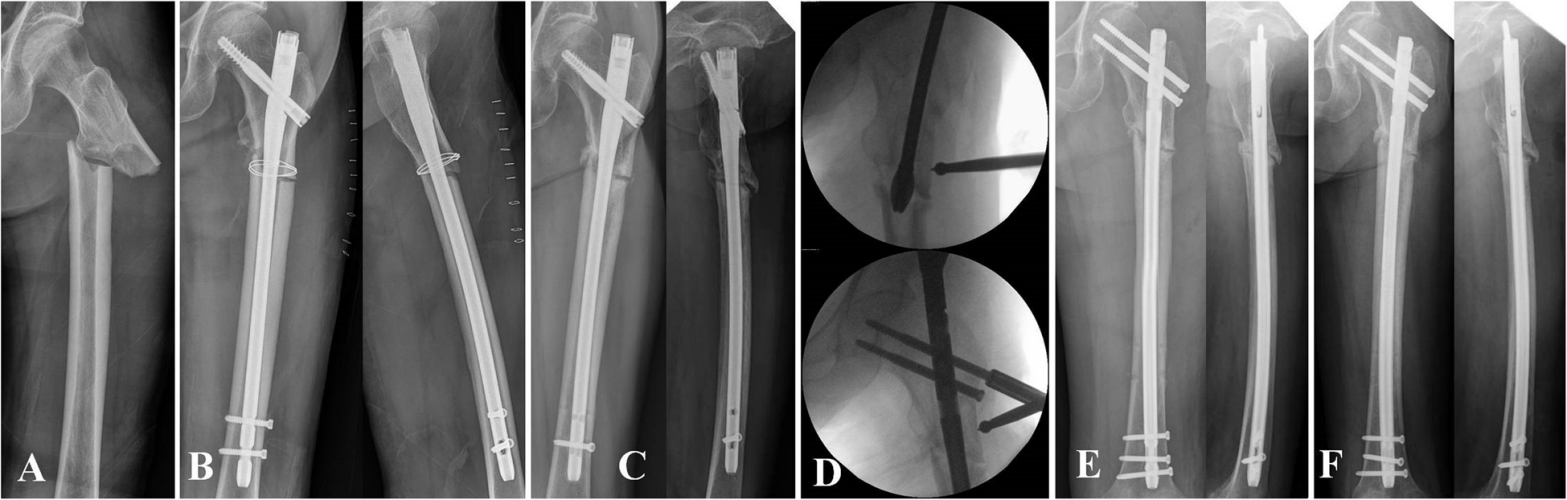
**a** A 68-year-old female patient who sustained atypical subtrochanteric fracture by low-energy injury. **b** Postoperative radiographs after open surgery. She underwent a total of three open surgeries. **c** Nonunion and implant failure at 43 months after initial surgery. **d** Revision surgery using closed procedure without bone graft. **e** Postoperative radiograph. **f** Nonunion at 1 year after revision surgery

We tested our hypothesis that the closed procedure without BG presents outcomes similar to those of the open procedure with BG in the treatment of subtrochanteric nonunion using the results of the current study. The results showed that union rate and time to union were not significantly different between the groups. Although the open procedure resulted in superior alignment correction than the closed one, the closed procedure yielded satisfactory results if the fracture gap was sufficiently reduced, even though alignment correction was less than that obtained with the open procedure. These results suggest that an open procedure with BG may not always be necessary for treating subtrochanteric nonunion. Furthermore, the BG procedure was not associated with the failure of revision surgery for subtrochanteric nonunion, but atypical femoral fracture, two or more previous surgeries, and angular malalignment were. In cases with atypical femoral fracture, two or more previous surgeries, large angular deformity before revision, and/or limitation in realignment during the revision surgery, further efforts such as open reduction and BG should be considered (Fig. 4).

Although there are relatively few reports on subtrochanteric nonunion, most of the literature considers BG as one of the important procedures for treating subtrochanteric nonunion. Haidukewych et al. [3] reported good surgical outcomes with revision internal fixation and selective BG for subtrochanteric nonunion. Barquet et al. [2] and von Rüden et al. [15] also reported good outcomes with revision surgery and selective BG for subtrochanteric nonunion. However, they did not specify the type of nonunion (hyper-, oligo-, or atrophic) or indications for BG. BG was performed according to the surgeon’s judgement. Lotzien et al. [16] and de Vries et al. [17] specified the type of nonunion in their literature; however, there were some cases in which the type of nonunion and BG procedure did not match. Although some patients in their studies showed atrophic nonunion, they did not undergo BG, while some who showed hypertrophic nonunion underwent BG. These points indicate that BG is performed according to the surgeon’s judgement based on clinical experience rather than definite indications, and no consensus has yet been established for the indication of BG. Thus, our study is significant in presenting a guideline for when BG should be considered for the surgical treatment of subtrochanteric nonunion.

**Fig. 5:**
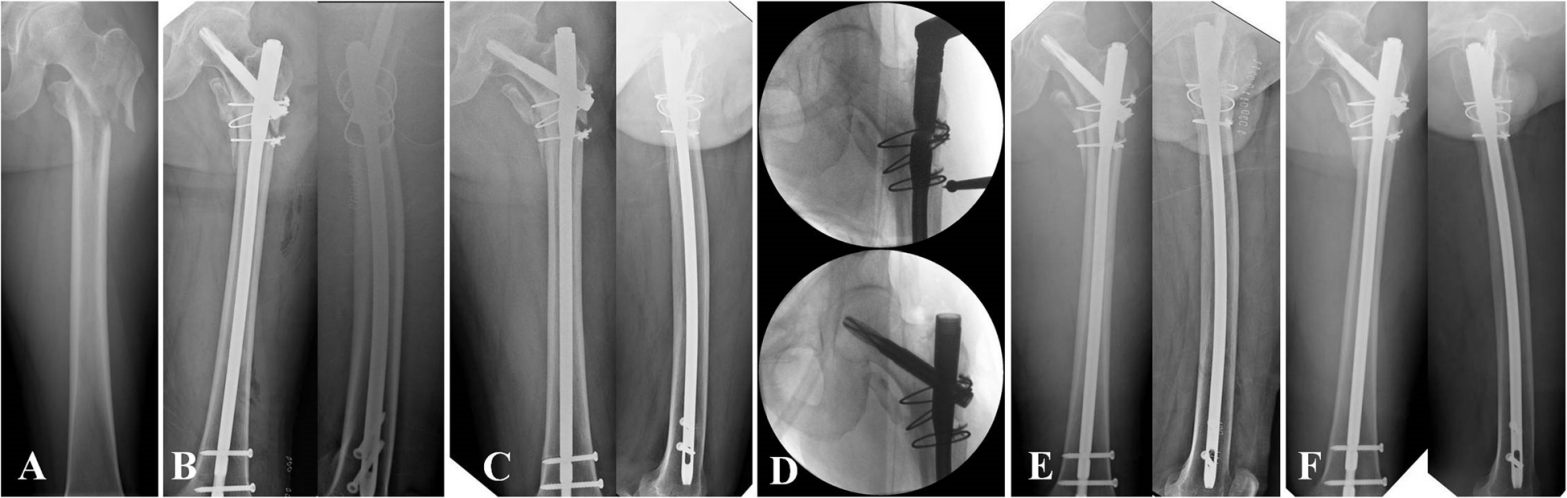
**a** A 44-year-old male patient. **b** Postoperative radiographs. **c** Nonunion and implant failure at 13 months after surgery. **d** Revision surgery using closed procedure without bone graft. **e** Postoperative radiographs. **f** Bony union at 7 months after revision surgery

The distinction between hyper-, oligo-, and atrophic nonunion is unclear clinically and practically. It cannot be assumed that every previous surgery, even if it was performed in an open manner, results in atrophic conditions. When there is a large fracture gap due to insufficient reduction and distraction in simple fracture, it may appear to be oligotrophic or atrophic nonunion due to excessive strain according to Perren’s strain theory [18], despite previous closed surgery. Nevertheless, when nonunion represents oligotrophic or atrophic conditions on radiological evaluation, BG is conventionally considered for the restoration of compromised biology. We believe that intramedullary reaming can reactivate the biological healing activity and work as an internal cancellous BG. Internal BG by intramedullary reaming has a long history [19, 20] and has been used clinically for the surgical treatment of femoral or tibial shaft nonunion [21, 22]. Previous studies by Wu et al. [9, 10] reported successful outcomes with the internal BG technique for the treatment of femoral shaft aseptic nonunion. They reported that internal BG can be performed with intramedullary reaming, and osteogenesis starts from inside out; moreover, the bone marrow is recanalized, and intramedullary vascularity can be established. Hence, we achieved satisfactory radiological results with the closed procedure and internal BG by intramedullary reaming without open BG (Fig. 5).

Elimination of the fracture gap by either compression between major fragments or open BG is one of the methods to improve the environment for fracture healing in surgery for nonunion [23]. However, providing complete compression between the fragments in revision surgery using a nail for subtrochanteric nonunion is not easy to perform technically and practically even with an open procedure because the fracture surface is not regular. Hence, we prefer using surgical methods such as the forward-striking technique to reduce the fracture gap and internal bone graft through intramedullary reaming to fill the remaining fracture gap.

This study aimed to confirm the radiological results of consecutive revision surgery for aseptic subtrochanteric nonunion through a closed procedure and reamed nailing without BG, which was not performed in previous studies. Satisfactory results could be achieved in this manner, and no significant differences in union rate and time to union were determined compared to the results of the open procedure with BG. Furthermore, the BG procedure was not associated with the failure of revision surgery, but atypical femoral fracture, two or more previous surgeries, and angular malalignment were. These results indicate that open BG is not always necessary when treating aseptic subtrochanteric nonunion. These significant findings provide a guideline for the indication of BG which has not yet been established clearly in subtrochanteric nonunion surgery, although further studies are required to provide stronger evidence.

The current study has several limitations, such as its retrospective nature and small sample size, resulting in an underpowered analysis and unavailability of a multivariate analysis and some factors in the univariate analysis. Therefore, future studies with large sample sizes and different study methods, such as meta-analyses or multi-center studies, are needed to overcome these limitations. Moreover, all the surgeries were performed by two surgeons in two institutions. Although the two surgeons were experienced, there could be a potential bias. However, considering that cases of subtrochanteric nonunion are rare and that there are relatively few studies regarding subtrochanteric nonunion and BG, the results of our study with follow-up of consecutive patients are significant.

## Conclusion

The radiological findings of revision surgery with percutaneous reduction and closed reamed nailing without BG for subtrochanteric nonunion were satisfactory. In the effort of percutaneous correction of malalignment, fracture gap reduction, and sufficient intramedullary reaming, the radiological results of the closed procedure without BG were not different from those of open nailing with BG, and the closed procedure without BG may be an acceptable option in appropriately selected patients. However, regarding the limitations of percutaneous reduction, specifically in flexion deformity, and the risk of nonunion in cases with atypical fracture and two or more previous surgeries, further efforts including an open procedure and BG should be considered. Further clinical studies with a large sample size are required to provide strong evidence.

## Data Availability

The datasets used and/or analysed in this study are available from the corresponding author upon reasonable request.

## Abbreviations

BG: Bone graft
AP: Anteroposterior
OR: Odds ratio
